# Dendritic Cells as Vaccines: Key Regulators of Tolerance and Immunity

**DOI:** 10.1155/2016/5789725

**Published:** 2016-05-31

**Authors:** Jurjen Tel, Daniel Benitez-Ribas, Edith M. Janssen, Evelien L. J. Smits, Joannes F. M. Jacobs

**Affiliations:** ^1^Department of Tumor Immunology, Radboudumc, Nijmegen, Netherlands; ^2^Enfermedades Hepaticas y Digestivas (CIBER), Barcelona, Spain; ^3^Division of Immunobiology, Cincinnati Children's Hospital Medical Center, University of Cincinnati, Cincinnati, OH, USA; ^4^Center for Oncological Research and Laboratory of Experimental Hematology, University of Antwerp, Antwerp, Belgium; ^5^Department of Laboratory Medicine, Radboudumc, Nijmegen, Netherlands

Dendritic cells (DCs) are a specialized family of professional antigen presenting cells that serve as a bridge linking the innate and adaptive arms of our immune system. DCs sense pathogens or interact with harmless antigens or nonpathogenic bacteria thereby tightly regulating the balance between tolerance and immunity. Despite their indispensable role in eliciting immune responses, DCs are a rather rare and heterogeneous type of immune cell, which differ in phenotype and function depending on maturation status, subsets, and age as well as their localization and microenvironment. Although scarce in numbers, cultured or naturally occurring DCs have been extensively investigated in clinical trials for both their capacity of priming antigen specific cytotoxic and helper T cells and humoral responses and their potential to induce immunological memory, which are capacities that distinct them from other, nowadays, exploited forms of immunotherapy.

Cancer immunotherapy has been designated the scientific breakthrough of the year in 2013. This has a broader implication for DC research in general, as DC based therapy can also be used to induce tolerance in autoimmune or immune-based diseases or to induce or improve immunity in, for instance, virally infected individuals. In this special issue we present two original research articles as well as five review papers on the therapeutic potential of the use of DC subsets for DC based immunotherapy in cancer, autoimmune disorders, and infectious diseases.

In their paper “Linking CD11b^+^ Dendritic Cells and Natural Killer T Cells to Plaque Inflammation in Atherosclerosis” M. Rombouts et al. performed extensive immune profiling in mice to investigate risk factors for plaque inflammation during atherosclerosis. They demonstrate that circulating CD11b^+^ cDC and NKT cells show great potential to reflect the inflammatory status in the atherosclerotic plaque. This may provide biomarkers with which atherosclerotic lesion progression can be monitored and may provide leads for immune cell based interventions.

J. Klarquist et al. provide an oversight of the changes in DC composition, maturation, and functionality in patients with systemic lupus erythematosus (SLE) and mouse models of spontaneous SLE. Based on the similarities between human and murine DC subsets as well as their reported relevance to disease, they suggest that mouse models provide a useful platform for the identification, dissection, and targeting of the DC intrinsic and extrinsic processes that facilitate the development, progression, and possibly a cure for SLE.

In the paper entitled “Immunity and Tolerance Induced by Intestinal Mucosal Dendritic Cells”, J. Aliberti describes the tolerogenic potential of DC in the digestive tract under steady-state conditions. The various DC subsets orchestrate tolerogenic responses towards commensal gut flora and they orchestrate powerful immune responses directed against invading pathogens. Failure to successfully complete this task may result in inflammatory bowel disease, food allergy, or celiac disease. Insight into the various DC subsets in the gut and the factors that influence their function may provide novel druggable targets as a basis for novel therapies.

Immunological tolerance remains a challenge in clinical organ transplantation and in management of autoimmune diseases. Tol-DCs are being regarded as a powerful tool to induce immune homeostasis in autoimmune diseases and as such are currently explored in clinical trials. In the review entitled “Metabolism Is Central to Tolerogenic Dendritic Cell Function” W. J. Sim et al. provide a thorough overview of how metabolic reprogramming of DCs drives differential cellular function and how this specifically contributes to pathologies. Furthermore, they describe and link tolerogenic DCs with immunosuppressive cytokines, for example, IL-10, and how these drive the shift in metabolism during TLR stimulation. Finally, they provide an overview on how pharmacological manipulation of the DC metabolism can be exploited for the generation of DC vaccines.

As the field of tolerogenic DC treatments moves forward, the need has arisen for the development of standardized protocols for the generation and application of DCs to allow comparison between different treatments and streamline the time from bench to bedside. A. T. Brinke et al. outline the efforts of the European A FACTT (Action to Focus and Accelerate Cell Based Tolerance Inducing Therapies) network that aims to harmonize DC production protocols, functional quality control parameters, immune monitoring parameters, and therapeutic regulations in order to accelerate the implementation of cell based tolerance inducing therapies in the clinic.

Currently, blood DC subsets are explored for the first time in clinical trials for treating metastatic cancer patients. S. P. Sittig et al. probed the potential of blood DC subsets to polarize and stimulate T cells. They specifically compared human plasmacytoid DCs (pDCs), BDCA1^+^ myeloid DCs (mDCs), and BDCA3^+^ mDCs and their ability to respond to TLR ligation and prime naive CD4^+^ T helper cells in an allogenic antigen unspecific and autologous antigen specific fashion. Although they clearly observed differences in the activation profile of the distinct DC subsets, all activated DC subsets were efficient in eliciting the production of IFN-*γ* by naive CD4^+^ T helper cells. Their findings further establish all three human blood DCs, despite their differences, as promising candidates for immunostimulatory effectors in cancer immunotherapy.

In the review “Pathogen-Associated Molecular Patterns Induced Crosstalk between Dendritic Cells, T Helper Cells, and Natural Killer Helper Cells Can Improve Dendritic Cell Vaccination,” T. Oth et al. describe the optimization of ex vivo generated DC vaccines by using rationally designed combinations of interferon gamma and different pathogen-associated molecular patterns for maturation. In this way, a cellular interplay is stimulated between key players of the antitumor response, DC, T helper 1 cells, natural killer cells, and cytotoxic T cells. Activation of multiple effector cell types might be the key to curative cancer vaccination. In this regard, interleukin 12-p70 is an important factor that stimulates efficient immunity. Attention should be paid to the generation procedure of the cellular vaccine so that the DC will still be able to produce interleukin 12 following injection. The outcome of DC vaccination might probably be further enhanced by making it part of a combination therapy that combines immune activation with attacking the immunosuppressive tumor microenvironment.

In summary, this special issue illustrates the function of various DC subsets and their contribution to tissue homeostasis. A better comprehension of the DC subsets and the networks they operate on may provide novel biomarkers to diagnose, prognosticate, and monitor disease. In addition, it may provide insights into improving the effectiveness of DC based immunotherapy.

*Jurjen Tel* *Jurjen Tel* *Daniel Benitez-Ribas* *Daniel Benitez-Ribas* *Edith M. Janssen* *Edith M. Janssen* *Evelien L. J. Smits* *Evelien L. J. Smits* *Joannes F. M. Jacobs* *Joannes F. M. Jacobs*

